# Heterogeneous distribution of *BRAF/NRAS* mutations among Italian patients with advanced melanoma

**DOI:** 10.1186/1479-5876-11-202

**Published:** 2013-08-29

**Authors:** Maria Colombino, Amelia Lissia, Mariaelena Capone, Vincenzo De Giorgi, Daniela Massi, Ignazio Stanganelli, Ester Fonsatti, Michele Maio, Gerardo Botti, Corrado Caracò, Nicola Mozzillo, Paolo A Ascierto, Antonio Cossu, Giuseppe Palmieri

**Affiliations:** 1 Unit of Cancer Genetics, Institute of Biomolecular Chemistry (ICB), National Research Council (CNR), Traversa La Crucca 3, Baldinca Li Punti 07100, Sassari, Italy; 2 Institute of Pathology, University Hospital (AOU), Sassari, Italy; 3 National Tumour Institute “Fondazione Pascale”, Naples, Italy; 4 Department of Dermatology, University of Florence, Florence, Italy; 5 Institute of Pathology, University of Florence, Florence, Italy; 6 Skin Cancer Unit, Istituto Scientifico Romagnolo Tumori (IRST), Meldola, Italy; 7 Department of Oncology, University Hospital (AOU), Siena, Italy

**Keywords:** Malignant melanoma, *BRAF* gene, *NRAS* gene, Mutation analysis, Cancer genetic heterogeneity

## Abstract

**Background:**

Prevalence and distribution of pathogenetic mutations in *BRAF* and *NRAS* genes were evaluated in multiple melanoma lesions from patients with different geographical origin within the same Italian population.

**Methods:**

Genomic DNA from a total of 749 tumor samples (451 primary tumors and 298 metastases) in 513 consecutively-collected patients with advanced melanoma (AJCC stages III and IV) was screened for mutations in exon 15 of *BRAF* gene and, at lower extension (354/513; 69%), in the entire coding DNA of *NRAS* gene by automated direct sequencing. Among tissues, 236 paired samples of primary melanomas and synchronous or asynchronous metastases were included into the screening.

**Results:**

Overall, mutations were detected in 49% primary melanomas and 51% metastases, for *BRAF* gene, and 15% primary tumors and 16% secondaries, for *NRAS* gene. A heterogeneous distribution of mutations in both genes was observed among the 451 primary melanomas according to patients’ geographical origin: 61% *vs*. 42% (p = 0.0372) *BRAF*-mutated patients and 2% *vs*. 21% (p < 0.0001) *NRAS*-mutated cases were observed in Sardinian and non-Sardinian populations, respectively. Consistency in *BRAF/NRAS* mutations among paired samples was high for lymph node (91%) and visceral metastases (92.5%), but significantly lower for brain (79%; p = 0.0227) and skin (71%; p = 0.0009) metastases.

**Conclusions:**

Our findings about the two main alterations occurring in the different tumor tissues from patients with advanced melanoma may be helpful in improving the management of such a disease.

## Introduction

Melanoma is characterized by a high tendency to metastasize and a striking resistance to conventional therapies other than surgery [1,2]. Recently, kinase-targeted therapies and immunostimulatory antibodies or a combination of them have been successfully introduced into the treatment of melanoma [3-7]. From the pathogenetic point of view, melanoma is a complex disease that arises thorough activation of several crucial cell-signaling pathways [8,9]. A better comprehension of the molecular mechanisms underlying the development and progression of melanoma is valuable in assessing the different biological subset of patients to be addressed to the most appropriate therapy.

Among others, the *mitogen-activated protein kinase* (MAPK) signal transduction pathway, which includes the cascade of NRAS, BRAF, MEK1/2, and ERK1/2 gene products, plays a major role in the pathogenesis of melanoma [10-12]. A high frequency of somatic mutations in *NRAS* and *BRAF* genes has been reported in both nevi and cutaneous melanomas, suggesting that such alteration may represent early events in the development of melanocytic tumors [13-15]. Furthermore, melanomas on skin that have not been chronically exposed to sun usually carry either a mutated *NRAS* or mutated *BRAF* (somatic mutations in such genes have been reported as mutually exclusive) [14,16,17].

Recently, our group demonstrated the occurrence of quite similar rates of *BRAF-NRAS* mutations among different types of metastasis, with a high consistency between primary melanomas and lymph node or visceral metastases, in contrast with a significantly lower consistency between primary tumors and brain or skin metastases [18]. The aim of this study was to evaluate prevalence and distribution of pathogenetic mutations in *BRAF* and *NRAS* genes among melanoma patients with different geographical origin within the same Italian population. In particular, we compared the *BRAF*/*NRAS* mutation frequencies between patients originating from Sardinia, whose population is considered genetically homogeneous due to its high rate of inbreeding and the subsequent inheritance of many common genetic traits [19,20], and those originating from other parts of Italy, whose genetic background is markedly heterogeneous (like that in vast majority of the general populations from Western countries). Finally, we extended the investigation about the distribution of *BRAF*-*NRAS* mutations to a larger series of different melanoma tissues.

## Patients and methods

### Patients

Five hundred and thirty-two patients with histologically-proven diagnosis of advanced melanoma (disease stages III and IV, according to American Joint Committee on Cancer guidelines [21]) were included into the study. Among them, 19 cases were excluded due to tissue DNA degradation; the remaining 513 cases had primary (N = 313) or metastatic (N = 62) or both (N = 138) tumor tissue samples available for mutation analysis. Patients were enrolled consecutively between June 2008 and March 2013 from centers in Italy. To avoid bias, patients were included regardless of age of onset, cancer family history, and disease characteristics. Sardinian or non-Sardinian (including cases from the central and southern regions in Italy) origin was ascertained in all cases through genealogical studies (place of birth of all patients and their parents was carefully assessed in order to assign their geographical origin). About one-fifth of the present cohort (108 patients) had been tested for *BRAF* and *NRAS* somatic mutations previously [18].

Patients were informed about the study aims and limits, and provided written consent for the molecular analysis on their tissue samples. The study was reviewed and approved by the ethical review boards at participating centers.

### Samples

Formalin-fixed, paraffin-embedded (FFPE) tumor tissues were obtained from pathological archives. To improve sensitivity of nucleotide sequencing, the neoplastic portion of each tissue section was isolated in order to obtain tumor samples with at least 80% neoplastic cells. Histological classification - including Breslow thickness, Clark’s level, and disease stage at diagnosis - was confirmed by medical records, pathology reports, and/or review of pathological material.

### Mutation analysis

Genomic DNA was isolated from FFPE tumour tissues, using the QIAamp DNA FFPE tissue kit (QIAGEN Inc., Valencia, CA, USA). The full coding sequences and splice junctions of *NRAS* (exons 2 and 3), and the entire sequence of the *BRAF* exon 15 (nearly all pathogenetic mutations of *BRAF* have been detected at the kinase domain at this genomic level [10]) were screened for mutations. All samples included into the study were assessed for the quality of the purified DNA, in order to avoid that discrepant cases could arise from technical problems such as the insufficient sample quality.

Sequencing conditions as well as primer sets and PCR assay protocols were as previously described [18,22]. Briefly, sequencing analysis was conducted in duplicate - starting from two different tumor sections and performing two different PCR-based amplifications - and in both DNA strands for all samples. For discordant tumors, the sequence analysis was performed in triplicate - three different tumor sections and three different PCR-based amplifications, in order to avoid any chance of PCR artifacts. A nucleotide sequence was considered as valid when the quality value (QV) was higher than 20 (<1/100 error probability), using a reference sequence for each of the analyzed exons (2 and 3 for *NRAS*, 15 for *BRAF*). In this study, the QV average was 35 (range, 30–45; <1/1000-1/10,000 error probability).

### Statistical analysis

Presence of *BRAF* or *NRAS* mutations was statistically correlated with different variables (sex, age at diagnosis and anatomical site of the primary melanoma, geographical origin of the patient) using the Pearson's Chi-Square test. The exact coefficient for sample proportion analysis was performed to determine all significant parameters (below 0.05 level). All analyses were performed using the statistical package SPSS/7.5 per Windows.

## Results

### Patients and samples

Genomic DNA from 513 consecutively-collected patients with advanced melanoma (AJCC stages III and IV [21]) was screened for somatic mutations in the exon 15 of *BRAF* gene. For a large fraction of patients whose DNA was available (354/513; 69%), mutation analysis was also carried out in the entire coding sequences of *NRAS* gene. PCR products corresponding to the coding exons and intron-exon junctions were analyzed by direct sequencing using an automated approach.

Majority of patients included into the study were males (277/513; 54%) and presented a disease with lymph node involvement (AJCC stage III, 319/513; 62%); median age was 55 years, with a range from 21 to 89 years (Table 1). Considering the anatomical site of the primary melanomas, trunk was the most frequent location (trunk, 243 [47%]; limbs, 205 [40%]; head and neck, 54 [11%]; unknown, 11 [2%]); median Breslow thickness was 2.1 mm (range, 0.78-8.3 mm). About one third (192/513; 37%) of patients originated from Sardinia; the remaining patients were from other geographical areas within central and Southern parts of Italy (Table 1). No substantial difference was observed in patients’ characteristics between the Sardinian and the non-Sardinian series.

**Table 1:** Characteristics of analyzed patients

** Characteristics**	**Number of patients**	***%***
**Total analyzed**	**513**	
Males/Females	277/236	*54/46*
Median age (years)	55	
Range	21-89	
***AJCC stage***		
III	319	*62*
IV	194	*38*
***Primary site***		
Head and neck	54	*11*
Limbs	205	*40*
Trunk	243	*47*
Unknown	11	*2*
***Geographical origin***		
Sardinian	192	*37*
non-Sardinian	321	*63*

*Abbreviation: AJCC* American Joint Committee on Cancer.

**Figure 1:**
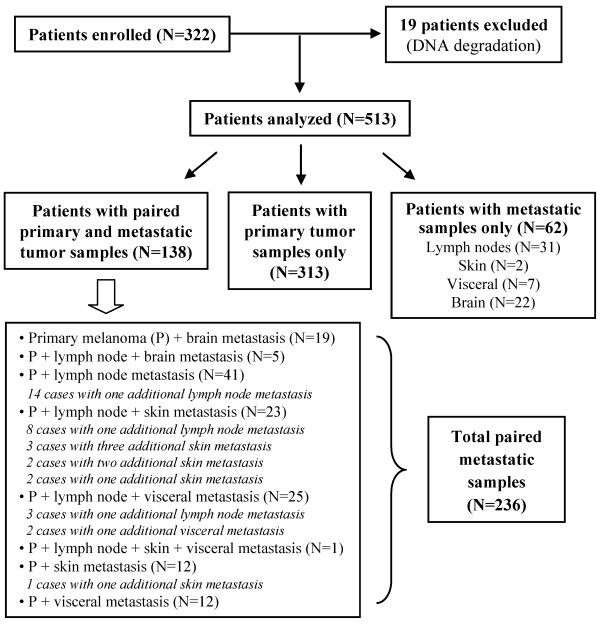
Patients and tissues included into the study.

Primary tumor tissues were the only available samples in a large fraction of patients (313/513; 61%). Among the remaining 200 patients, paired samples of primary melanomas and synchronous or asynchronous metastases were obtained from about one fourth of cases (138/513; 27%), whereas metastatic tumor tissues represented the only available specimens for about one tenth of cases (62/513; 12%) (Figure 1). Overall, a total of 749 tumor samples (451 primary melanomas and 298 melanoma metastases) was screened for *BRAF* mutations; among them, available DNA from 528 specimens (312 primary melanomas and 216 melanoma metastases) was analyzed for mutations in *NRAS* gene.

### Mutation analysis

Mutations in *BRAF* gene were detected in 223/451 (49%) primary melanomas and 153/298 (51%) metastatic tissues, whereas *NRAS* mutations were found in 46/312 (15%) primary tumors and 34/216 (16%) melanoma metastases (Table 2). In our series, no concomitant mutations of *BRAF* and *NRAS* genes were detected. Overall, *BRAF* or *NRAS* mutations were observed in 376/749 (50%) or 80/528 (15%) melanoma tissue samples, respectively (Table 2). Considering the cases analyzed for mutations in both genes, we identified a *BRAF* or *NRAS* mutation in 339/528 (64%) melanoma lesions.

**Table 2:** **Prevalence of *BRAF*/*NRAS *mutations in melanoma tissues**

**Type of sample**	***BRAF***	***NRAS***
	**mutated**	**mutated**
	**(*%*)**	**(*%*)**
**Primary tumour**	**223/451**	**46/312**
	**(49%)**	**(15%)**
Sardinian	109/178	2/105
patients	(61%)	(2%)
non-Sardinian	114/273	44/207
patients	(42%)	(21%)
**Metastasis**	**153/298**	**34/216**
	**(51%)**	**(16%)**
Lymph node	78/151	15/102
	(52%)	(15%)
Visceral	25/47	4/30
	(53%)	(13%)
* Liver*	*16/30*	*3/22*
	*(53%)*	*(14%)*
* Lung*	*9/17*	*1/8*
	*(53%)*	*(12%)*
Skin	29/54	5/38
	(54%)	(13%)
Brain	21/46	10/46
	(46%)	(22%)
**Total lesions**	**376/749**	**80/528**
	**(50%)**	**(15%)**

Among the metastatic tissue samples, with the exception of the brain metastases [*BRAF*, 21/46 (46%); *NRAS*, 10/46 (22%)], a quite identical frequency of *BRAF* and *NRAS* mutations was observed across the different types of metastasis: lymph nodes [*BRAF*, 78/151 (52%); *NRAS*, 15/102 (15%)], visceral lesions [*BRAF*, 25/47 (53%); *NRAS*, 4/30 (13%)], and subcutaneous lesions [*BRAF*, 29/54 (54%); *NRAS*, 5/38 (13%)] (Table 2).

According to the patients’ geographical origin, distribution of mutations among the 451 primary melanomas was significantly heterogeneous for both genes: for *BRAF*, 109/178 (61%) *vs*. 114/273 (42%) mutated patients were observed in Sardinian and non-Sardinian populations, respectively (p = 0.0372); for *NRAS*, 2/105 (2%) *vs*. 44/207 (21%) mutated cases were observed in Sardinian and non-Sardinian populations (p < 0.0001) (Table 2).

Nearly all *BRAF* mutations across samples were of the BRAF^V600E^ subtype (347/376; 92.3%). All but one of the remaining *BRAF* variants were represented by other V600 subtypes: V600K (20/376; 5.3%), V600D (6; 1.6%), and V600R (2; 0.5%) (Table 3). The L597R variant (1/376; 0.3%) was therefore the only mutation not affecting the codon 600 of *BRAF*, though it is a sequence variation still localized into the active kinase domain of the gene (Table 3). For *NRAS*, all deleterious mutations were found at the codon 61 of the gene: Q61R (47/80; 58.8%), Q61L (23; 28.7%), and Q61K (10; 12.5%) (Table 3). All mutations detected in this study have been reported previously in the Human Gene Mutation Database (HGMD) at http://www.hgmd.cf.ac.uk/ac/index.php and in the Catalogue Of Somatic Mutations In Cancer (COSMIC) at http://www.sanger.ac.uk/genetics/CGP/cosmic/.

**Table 3:** **Mutations in *BRAF*/*NRAS *genes**

**Exon**	**Mutation**	**Base change**	**Amino acid change**	**Mutated samples**	**%**
*BRAF*					
15	V600E	1799	Val to Glu	347	92.3
		T > A			
15	V600K	1798-99 GT > AA	Val to Lys	20	5.3
15	V600D	1799-1800	Val to Asp	6	1.6
		TG > AT			
15	V600R	1798-99	Val to Arg	2	0.5
		GT > AG			
15	L597R	1790	Leu to Arg	1	0.3
		T > G			
*NRAS*					
3	Q61R	182	Gln to Arg	47	58.8
		A > G			
3	Q61L	182	Gln to Leu	23	28.7
		A > T			
3	Q61K	181	Gln to Lys	10	12.5
		C > A			

Frequencies are related to the total amount of mutated cases in *BRAF* (N = 376) and *NRAS* (N = 80) genes.

Among the 236 paired samples of primary and secondary melanomas, 202 (86%) showed concordant mutation patterns between primary tumors and metastatic lesions. In particular, rates of consistency in *BRAF/NRAS* mutations between primary and secondary tumors varied according to the anatomical sites of metastasis: 109/120 (91%; p = 0.1123), for metastases in lymph nodes; 37/40 (92.5%; p = 0.4316), for visceral metastases; 19/24 (79%; p = 0.0227), for brain metastatic lesions; 37/52 (71%; p = 0.0009), for skin secondaries (Table 4). Synchronous metastases (though they represented a limited fraction of the total amount of secondary lesions) showed a slight, non-significant higher rate of consistency in comparison to that found among asynchronous metastases [40/44 (91%) vs. 162/192 (84%), respectively] (Table 4).

**Table 4:** **Consistency between primary and secondary melanoma lesions, according to the *BRAF/NRAS *mutation status**

**Tissue types**	**Consistency secondary/primary melanomas *(%)***	**Synchronous metastases**	**Asynchronous metastases**
Lymph node	109/120	25/26	84/94
metastasis	*(90.8%)*	*(96.2%)*	*(89.4%)*
Visceral metastasis	37/40	3/3	34/37
	*(92.5%)*	*(100%)*	*(91.9%)*
Brain	19/24	1/1	18/23
metastasis	*(79.2%)*	*(100%)*	*(78.3%)*
Skin	37/52	11/14	26/38
metastasis	*(71.2%)*	*(78.6%)*	*(68.4%)*
TOTAL	202/236	40/44	162/192
	*(85.6%)*	*(90.9%)*	*(84.4%)*

Considering the 34 paired samples with discrepancies in *BRAF/NRAS* mutation patterns between primary and secondary tumors, majority of them (18; 53%) displayed a wild-type primary tumor and a mutated metastasis (14 in *BRAF* and 4 in *NRAS*), a second large fraction of cases (14/34; 41%) presented with a mutated primary tumor and a wild-type metastasis (13 in *BRAF* and 1 in *NRAS*), and the remaining limited subgroup of samples (2/34; 6%) carried a change in mutation pattern between the two tumor lesions (an *NRAS* mutation in primary melanoma and a *BRAF* mutation in melanoma metastasis) (Table 5).

**Table 5:** Mutation patterns in discrepant cases

**Tissue types**	**Discrepancy secondary/primary melanomas *(%)***	**Primary tumour**		**Metastasis**	
		***BRAF***	***NRAS***	***BRAF***	***NRAS***
Lymph node metastasis	11/120	**V600K**	wt	wt	wt
		wt	wt	**L597R**	wt
		wt	wt	**V600E**	wt
		wt	wt	**V600E**	wt
		wt	wt	**V600E**	wt
	*(9.2%)*	**V600E**	wt	wt	wt
		wt	wt	**V600E**	wt
		**V600E**	wt	wt	wt
		**V600E**	wt	wt	wt
		wt	wt	**V600E**	wt
		**V600E**	*not tested*	wt	*not tested*
Visceral metastasis	3/40	**V600E**	wt	wt	wt
		wt	wt	**V600E**	wt
	*(7.5%)*				
		wt	wt	**V600E**	wt
Brain metastasis	5/24	**V600E**	wt	wt	wt
		wt	wt	**V600E**	wt
		wt	wt	wt	**Q61L**
	*(20.8%)*				
		wt	wt	wt	**Q61L**
		wt	wt	wt	**Q61R**
Skin	15/52	wt	wt	wt	**Q61L**
		**V600E**	wt	wt	wt
		**V600E**	wt	wt	wt
		wt	**Q61R**	wt	wt
		wt	**Q61R**	**V600E**	wt
		wt	**Q61R**	**V600E**	wt
metastasis	*(28.8%)*				
		wt	wt	**V600E**	wt
		wt	wt	**V600E**	wt
		**V600E**	wt	**V600E**	wt
		wt	wt	**V600E**	wt
		wt	wt	**V600E**	wt
		**V600E**	wt	wt	wt
		wt	wt	**V600E**	wt
		**V600E**	*not tested*	wt	*not tested*
		**V600E**	*not tested*	wt	*not tested*

*Abbreviation: wt* wild-type.

With the exception of the age at diagnosis, the frequency of *BRAF* mutations was not correlated with any clinicopathological parameters in primary melanomas. The prevalence of *BRAF* mutations was significantly higher in patients with onset age of 50 years or younger (101/166; 60.8%) as compared with those older than 50 years (122/285; 42.8%) (p = 0.0431). No correlation was instead observed between *NRAS* mutations and clinicopathological parameters. The *BRAF/NRAS* mutation status was not evaluated for association with clinical outcome in our series.

## Discussion

The *NRAS* and *BRAF* genes encode two important proteins belonging to the *mitogen-activated protein kinase* (MAPK) signal transduction pathway, which regulates cell growth, survival, and invasion [12,23,24]. Mutations in these genes have been widely implicated in several aspects of development and progression of melanoma [25,26]. In the present study, we evaluated the spectrum and distribution of somatic mutations in *NRAS* and *BRAF* genes in a large series of melanoma tissues (N = 749, including 451 primary melanomas and 298 melanoma metastases), excised from patients with different geographical origin within the Italian population.

Overall, *BRAF* mutations were observed in half of our tissue sample collection (376/749; 50.2%), whereas *NRAS* mutations were detected in about one seventh of analyzed cases (80/528; 15.1%). Since *BRAF* and *NRAS* mutations were found to be mutually exclusive (further confirming previous data [23]), a high prevalence of such alterations was observed in our series, with about two thirds of melanomas presenting a *BRAF/NRAS* mutation. All detected *BRAF* or *NRAS* variants have been previously demonstrated to be oncogenic and able to induce constitutive ERK activation, which in turn promotes cell proliferation and survival. With the exception of the *BRAF*^L597R^ variant, all mutations occurred in codons V600 and Q61 of *BRAF* and *NRAS* genes, respectively (see Table 3). While the rates of the mutation subtypes in *NRAS* gene were comparable with those described in majority of previous reports, the BRAF^V600E^ mutation represented the most preponderant *BRAF* variant in our series (92.3%), with an incidence of the other BRAF^V600^ mutation subtypes much lower (about 7%) than that reported in Australian population (ranging from 26% to 30%) [27-29].

Considering the different types of metastatic lesions, rates of *BRAF* and *NRAS* mutations were highly similar across the lymph node (52%, for *BRAF*, and 15%, for *NRAS*), visceral (53% and 13%), and skin (54% and 13%) metastases (see Table 2). Although the total amount of *BRAF*/*NRAS* mutated cases was quite identical to that of the other secondary lesions, brain metastases surprisingly presented a markedly divergent distribution of *BRAF* (46%) and *NRAS* (22%) mutations.

A quite similar frequency of either *BRAF* or *NRAS* mutations was observed among primary and metastatic melanomas: 49% *vs*. 51%, for *BRAF*, and 15% *vs*. 16%, for *NRAS*, respectively (see Table 2). On this issue some controversial data have been provided. The lack of a significant difference in *BRAF*/*NRAS* mutations between primary and secondary melanomas in our series seems to be consistent with previous data indicating that *BRAF*/*NRAS* mutations may occur early in the development of melanoma and, therefore, their incidence may not vary significantly during tumor progression [30,31]. Furthermore, the presence of *BRAF* mutations in nevi [13-15] suggests that activation of the RAS/RAF/MEK/ERK pathway may participate to initiation of melanocytic transformation as well as that *BRAF* activation is necessary for inducing cell proliferation but not sufficient for the development of melanoma (additional molecular events are thus required to achieve full malignancy). Conversely, the demonstration of a sequential increase in mutation rates for both *BRAF* and *NRAS* genes in a subset of melanomas during progression of the disease - from in-situ to invasive melanomas [32,33] or from primary to metastatic melanoma lesions and melanoma cell lines in a more limited series previously analyzed by our group [18] - also suggests that *BRAF*/*NRAS* mutations can not be strictly considered as founder events in melanomagenesis for the totality of cases (in truth, a slightly increased incidence of such mutations, moving from primary to metastatic lesions, was indeed registered in this study).

A significantly higher frequency of *BRAF* mutations was present in primary melanoma patients from Sardinia, as compared to those from the other parts of Italy (61% *vs*. 42%; p = 0.0372), whereas a significantly higher prevalence of *NRAS* mutations was found in cases from Middle-South Italy, as compared to those from Sardinia (21% *v*s. 2%; p < 0.0001). The Sardinian population (1.67 million in 2010, according to the Italian National Institute of Statistics) is considered genetically homogeneous, since it is isolated and has experienced little immigration due to its remote location [19,20]; conversely, the remaining Italian populations are genetically heterogeneous and similar to the mixed ones into the Western countries. Our observations strongly suggest that different “genetic background” may induce discrepant “penetrance” and distribution of somatic mutations in candidate cancer genes. On this regard, one could speculate that mechanisms of transformation underlying the pathogenesis of melanoma may differ in distinct populations. This represents a further confirmation of previous results on germline DNA from different collections of melanoma patients, indicating that genetic factors involved in susceptibility to melanoma are geographically heterogeneous and strictly dependent on patients’ origin [34,35].

It is worthy to underline that the total amount of *BRAF*/*NRAS* mutated cases was however identical among the Sardinian (61%, for *BRAF*, and 2%, for *NRAS*; total frequency, 63%) and non-Sardinian (42%, for *BRAF*, and 21%, for *NRAS*; total frequency, 63%) patients in our series. These findings seem to suggest that the MAPK pathway may be activated - through occurrence of either *BRAF* or *NRAS* mutations - in a maximal fraction of about two thirds of melanoma cases. While the different mutation frequencies in such cancer genes could be explained by differences into the genetic background related to distinct patients’ origin, there is no clear explanation about the putative existence of a limit in rates of oncogenic activation of the MAPK pathway. Prospectively, evaluation of a larger collection of data from melanoma series screened worldwide for somatic mutations in both genes may provide additional clues about this issue. Nevertheless, our findings indicate that the mutation prevalence for any candidate cancer gene needs to be accurately assessed in each geographical area.

Thirty-four paired samples (14.4%) out of 236 analyzed demonstrated discrepancies in *BRAF/NRAS* mutation patterns between primary and secondary tumors; a significant discrepancy was only observed in subcutaneous (28,8%; p = 0.0009) or cerebral metastases (20.8%; p = 0.0227). Although at a non-significant rate, discrepancies were more frequent in asynchronous than synchronous metastases (15.6% vs. 9.1%, respectively). In half of the discrepant cases, we found a wild-type primary tumor and a mutated metastasis (78% *BRAF* and 22% *NRAS*). In the remaining discrepant cases, we surprisingly observed a mutated primary tumor and a wild-type metastasis (93% *BRAF* and 7% *NRAS*) or, to a less extent, a different mutation pattern between melanoma lesions (*NRAS* mutation in primary and *BRAF* mutation in secondary tumors) (see Table 5). While for the first half of the discrepant cases, one could infer that selection of *BRAF*/*NRAS* mutant alleles may occur during tumor progression, for the second series, one could speculate that primary melanoma may be heterogeneous with different tumor cell types (one mainly represented and the others less represented, which may be able to however give origin to metastatic subclones in a subset of cases). In this sense, molecular heterogeneity as well as polyclonality of *BRAF* mutations in primary melanomas have been widely reported [33,36,37].

Although pathogenetic mechanisms underlying melanoma development and progression are multiple and still largely unknown, classification of melanoma patients through the assessment of the molecular profile in primary tumors and/or correspondent metastases is becoming mandatory. In clinical practice, our future efforts will be aimed at unveiling which gene or pathway could be truly affected in which subset of patients, in order to achieve the best treatment and management of the disease. With the present study, we provided additional clues about the spectrum and distribution of the two main alterations frequently occurring in the different tumor tissues from patients with advanced cutaneous melanoma.

## Abbreviations

COSMIC: Catalogue of somatic mutations in cancer; FFPE: Formalin-fixed paraffin-embedded; HGMD: Human gene mutation database; MAPK: Mitogen-activated protein kinase; PCR: Polymerase chain reaction.

## Competing interest

PAA is consultant of Bristol Myers Squibb, MSD, and Roche-Genentech. He participated into the Advisory Board from Bristol Myers Squibb, MSD, Roche-Genentech, GSK, Amgen, Celgene, Medimmune, and Novartis. He received honoraria from Brystol Myers Squibb, MSD, and Roche-Genentech. All remaining authors declare the absence of any conflict of interest.

## Authors’ contributions

MCo, performed mutation analysis and data interpretation, helped to draft the manuscript; AL, performed quality control of pathological data; MCa, performed data analysis; VDG, DM, IS, EF, MM, GB, CC, NM, and PAA participated in patients’ collection and data acquisition; AC, performed pathological review and data analysis, participated into the design of the study; GP, performed data interpretation, conceived of the study, drafted the manuscript. All authors read and approved the final manuscript.
